# Supplementary material for: The MYST Family Histone Acetyltransferase SasC Governs Diverse Biological Processes in Aspergillus fumigatus
Source: Cells. 2023 Nov 16;12(22):2642. doi: 10.3390/cells12222642 (PMC10670148; doi:10.3390/cells12222642)
Supplement: Supplementary file 1 [file cells-12-02642-s001.zip › Figure S1.pptx]

## Slide 1
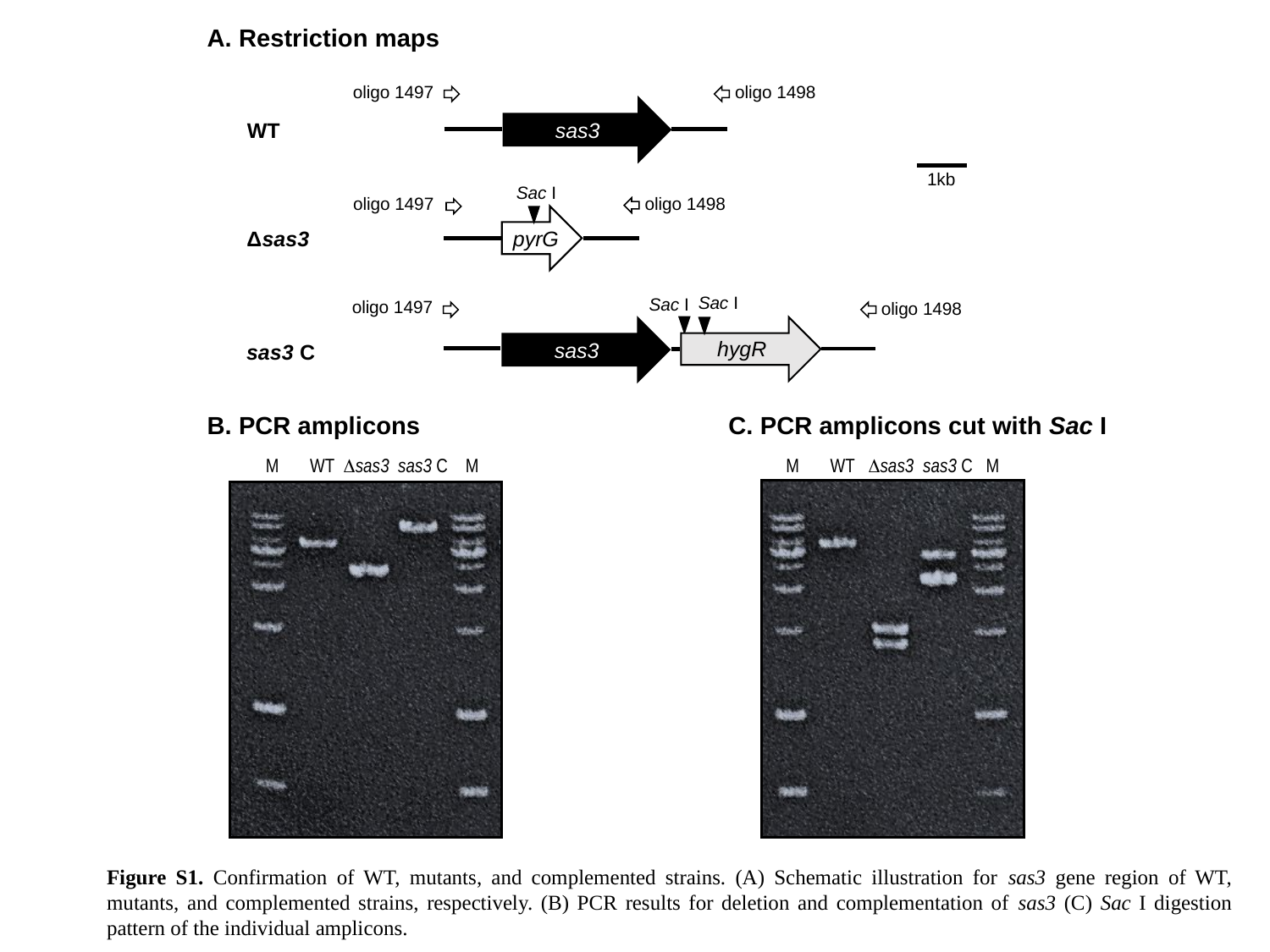

A. Restriction maps
oligo 1497
oligo 1498
sas3
WT
1kb
Sac I
oligo 1497
oligo 1498
pyrG
Δsas3
Sac I
Sac I
oligo 1497
oligo 1498
hygR
sas3
sas3 C
B. PCR amplicons
M WT Dsas3 sas3 C M
C. PCR amplicons cut with Sac I
M WT Dsas3 sas3 C M
Figure S1. Confirmation of WT, mutants, and complemented strains. (A) Schematic illustration for sas3 gene region of WT, mutants, and complemented strains, respectively. (B) PCR results for deletion and complementation of sas3 (C) Sac I digestion pattern of the individual amplicons.
