# Supplementary material for: The MYST Family Histone Acetyltransferase SasC Governs Diverse Biological Processes in Aspergillus fumigatus
Source: Cells. 2023 Nov 16;12(22):2642. doi: 10.3390/cells12222642 (PMC10670148; doi:10.3390/cells12222642)
Supplement: Supplementary file 1 [file cells-12-02642-s001.zip › FIgure S2.pptx]

## Slide 1
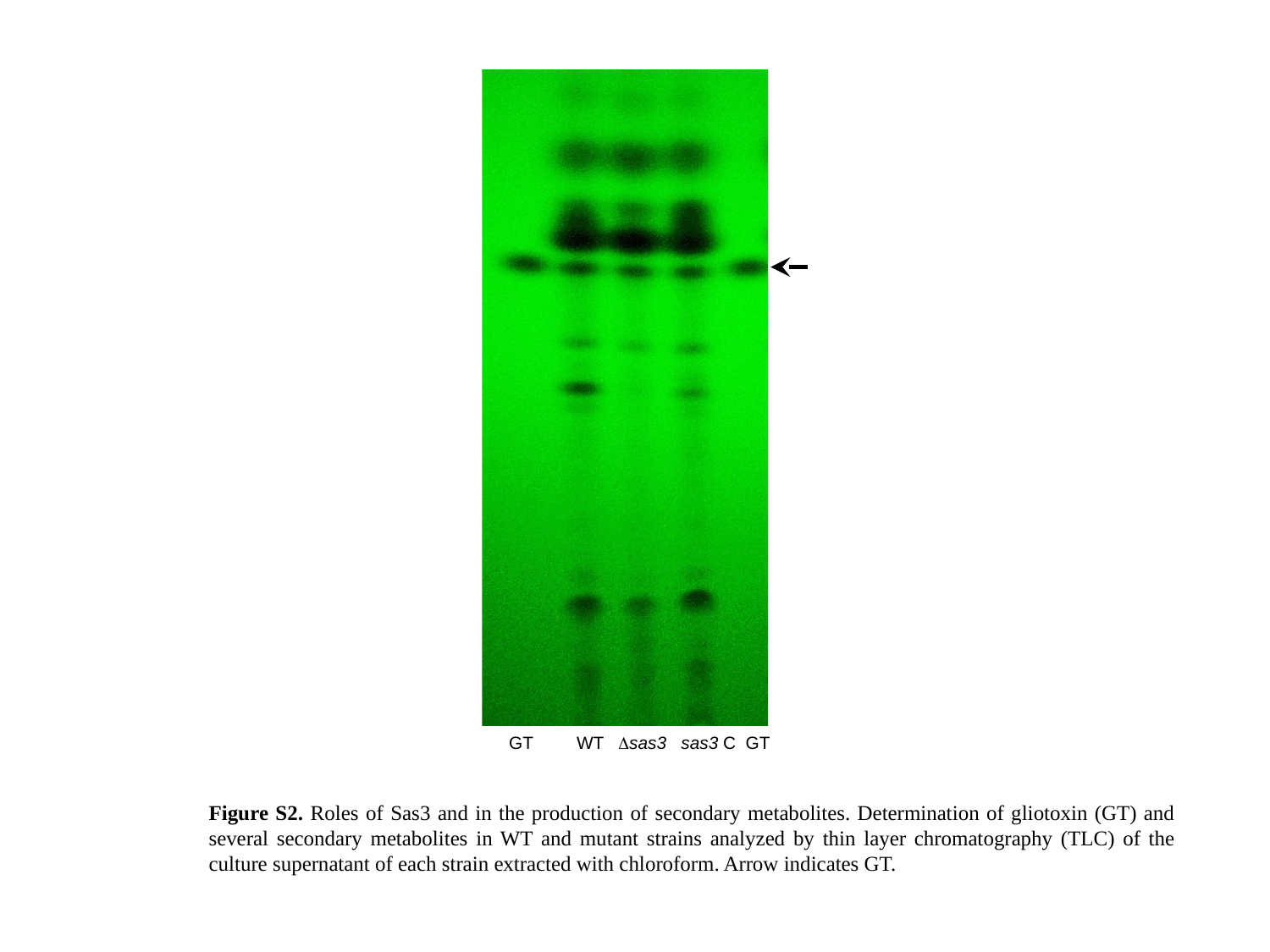

GT WT Dsas3 sas3 C GT
Figure S2. Roles of Sas3 and in the production of secondary metabolites. Determination of gliotoxin (GT) and several secondary metabolites in WT and mutant strains analyzed by thin layer chromatography (TLC) of the culture supernatant of each strain extracted with chloroform. Arrow indicates GT.
