# Supplementary material for: The MYST Family Histone Acetyltransferase SasC Governs Diverse Biological Processes in Aspergillus fumigatus
Source: Cells. 2023 Nov 16;12(22):2642. doi: 10.3390/cells12222642 (PMC10670148; doi:10.3390/cells12222642)
Supplement: Supplementary file 1 [file cells-12-02642-s001.zip › FIgure S3.pptx]

## Slide 1
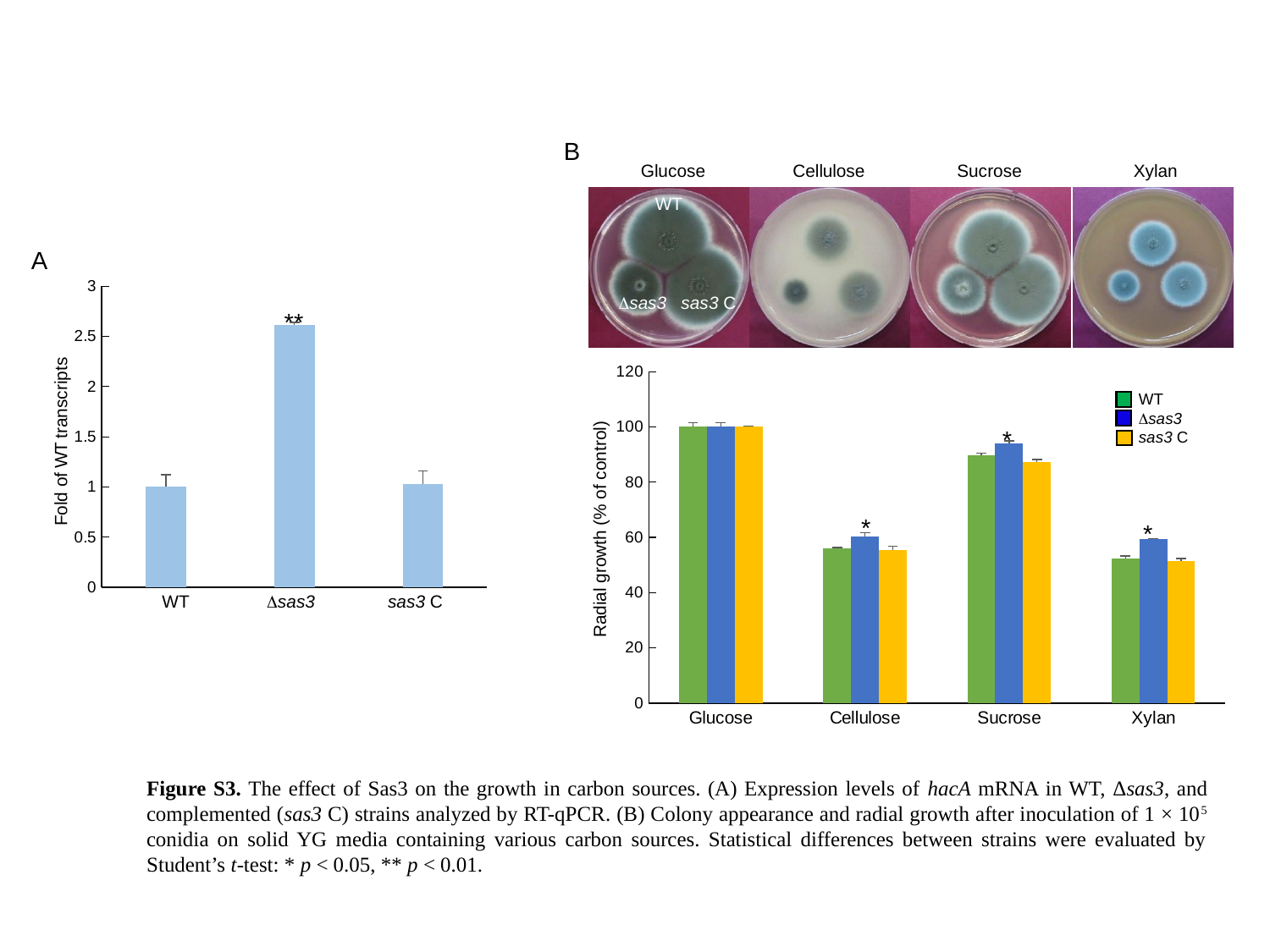

B
 Glucose Cellulose Sucrose Xylan
WT
Dsas3 sas3 C
### Chart
| Category | | | |
|---|---|---|---|
| Glucose | 100.0 | 100.0 | 100.0 |
| Cellulose | 55.893376632162905 | 60.4404862197617 | 55.48026245025277 |
| Sucrose | 89.61234016642987 | 94.1228386889718 | 87.37227062493277 |
| Xylan | 52.229213179081256 | 59.3573233842821 | 51.41581059199083 |*
*
*
Radial growth (% of control)
WT
Δsas3
sas3 C
A
### Chart
| Category | |
|---|---|**
Fold of WT transcripts
 WT Dsas3 sas3 C
Figure S3. The effect of Sas3 on the growth in carbon sources. (A) Expression levels of hacA mRNA in WT, Δsas3, and complemented (sas3 C) strains analyzed by RT-qPCR. (B) Colony appearance and radial growth after inoculation of 1 × 10^5^ conidia on solid YG media containing various carbon sources. Statistical differences between strains were evaluated by Student’s t-test: * p < 0.05, ** p < 0.01.
