# Supplementary material for: The MYST Family Histone Acetyltransferase SasC Governs Diverse Biological Processes in Aspergillus fumigatus
Source: Cells. 2023 Nov 16;12(22):2642. doi: 10.3390/cells12222642 (PMC10670148; doi:10.3390/cells12222642)
Supplement: Supplementary file 1 [file cells-12-02642-s001.zip › Table S1.pdf]

Table S1. Oligonucleotides used in this study.

Name	Sequence (5'→3')[a]	Purpose
oligo 256	TTCCAAGCAGAGCTTGTCAC	5' *brlA* for qRT-PCR
oligo 257	CCAGGTTCTTTGCACTTGAA	3' *brlA* for qRT-PCR
oligo 303	GCTACCACTCTGCATCCTCA	5' *abaA* for qRT-PCR
oligo 304	TACGAGCTCCAGCATGATTC	3' *abaA* for qRT-PCR
oligo 305	ACGGCAGGAAGTTGTCTTCT	5' *wetA* for qRT-PCR
oligo 306	CTGTCAGCGACTTGTTGGAT	3' *wetA* for qRT-PCR
oligo 346	CCATGTGTGTCGAGTCCTTC	5' *ef1* for qRT-PCR normalization
oligo 347	GAACGTACAGCAACAGTCTGG	3' *ef1* for qRT-PCR normalization
oligo 430	TTCCAAATGTGGCAAGTGAT	5' *acyA* for qRT-PCR
oligo 431	GCAAACGTGGAATCAATACG	3' *acyA* for qRT-PCR
oligo 434	CCACCACCTACAACAACAGC	5' *pkaC1* for qRT-PCR
oligo 435	TGTGAAGACGCATGATGAGA	3' *pkaC1* for qRT-PCR
oligo 1260	TAGCGGAAATGCCAACGGAGGATA	5' *chsB* for qRT-PCR
oligo 1261	GAGAAGCAGGGCGCGATGAATACT	3' *chsB* for qRT-PCR
oligo 1262	ATCGCTGCATTCCTGGGTCTGTCG	5' *chsE* for qRT-PCR
oligo 1263	CGCGCAAGCTCATCGGCATTTT	3' *chsE* for qRT-PCR
oligo 1493	CGCATCGAGCTGACGATATG	5' flanking region of *sas3*
oligo 1494	*CTGATCTACCCCTTGGAACGCAGCA*TCGCCGTAGTCGCCAGTTT	3' *sas3* with *AnpyrG* tail

oligo 1495	*TTTGTAGGCTTTGGGCTGTTCACAA*GACAACCAGATAGGAAACGG	5' *sas3* with *AnpyrG* tail
oligo 1496	CTATGTACGGTGTGACCTTAG	3' flanking region of *sas3*
oligo 1497	ACCTTCCATGGGTTGCTGAC	5' nested of *sas3*
oligo 1498	ATGTATACTCTGCACTGCCC	3' nested of *sas3*
oligo 1667	GTGCGCGCTATCCAAACAAT	5' *gfaA* for qRT-PCR
oligo 1668	ACGACTGACTCGGGTTGTTC	3' *gfaA* for qRT-PCR
oligo 1697	*TAGTTCTGTTACCGAGCCGG*CCGTTTCCTATCTGGTTGTC	5' *sas3* with *hygB* tail for C'
oligo 1698	*GCTCTGAACGATATGCTCCAAC*AGGGCAAAGCACGAACGGTA	3' *sas3* with *hygB* tail for C'
oligo 1699	ACTCCCTCCACTCCTGTTGT	5' *ire1* for qRT-PCR
oligo 1700	CGCCTTCTTGTTGCTCTTCG	3' *ire1* for qRT-PCR
oligo 1701	CGGACTCGAAGGACCTGGAA	5' *hacA* for qRT-PCR
oligo 1702	TAGGCGGTGGAAGTCATCGT	3' *hacA* for qRT-PCR
oligo 1703	CCCGTGCAAGTCTCACCAAA	5' *musN* for qRT-PCR
oligo 1704	TTGCTGATTGCACAGGCGAT	3' *musN* for qRT-PCR
oligo 1705	GTCCCTCTTGCTCTACCGT	5' *uve1* for qRT-PCR
oligo 1706	TCTTCGGTCGAAGCCACTCT	3' *uve1* for qRT-PCR

[a] Tail sequence is in italic.
