# Supplementary material for: The MYST Family Histone Acetyltransferase SasC Governs Diverse Biological Processes in Aspergillus fumigatus
Source: Cells. 2023 Nov 16;12(22):2642. doi: 10.3390/cells12222642 (PMC10670148; doi:10.3390/cells12222642)
Supplement: Supplementary file 1 [file cells-12-02642-s001.zip › Table S2.pdf]

Table S2. Differentially regulated genes response to stimuli (Fold change > 2.0, *p*-value < 0.01).

Gene symbol	Fold change	*p*-value	Product
2G05060	42.734	0.001	alternative oxidase AlxA, putative
6G04670	10.830	0.000	ER-associated proteolytic system protein Der1, putative
4G11310	9.925	0.004	fructose-1,6-bisphosphatase Fbp1, putative
3G12270	9.780	0.000	glutathione peroxidase Hyr1, putative
2G11070	9.087	0.000	tyrosyl-DNA phosphodiesterase, putative
5G05640	8.266	0.006	RTA1 domain protein, putative
3G07940	7.516	0.000	phosphoinositide-specific phospholipase C, putative
5G02300	7.228	0.008	peroxidase, putative
3G10770	5.965	0.003	RTA1 domain protein, putative
4G03460	5.116	0.000	HLH DNA binding domain protein, putative
6G13340	5.045	0.004	mismatch-specific thymine-DNA glycosylase, putative
2G02090	4.879	0.000	methylated-DNA-protein-cysteine methyltransferase
4G10210	4.514	0.001	phospholipid-transporting ATPase (DRS2), putative
4G11140	4.389	0.001	DNA polymerase iota, putative
2G16520	4.184	0.002	phospholipase D (PLD), putative
2G15130	3.633	0.000	ABC multidrug transporter, putative
1G14330	3.627	0.003	ABC transporter, putative
1G01720	3.615	0.002	protein kinase and ribonuclease Ire1, putative
6G07700	3.582	0.005	chromosomal organization and DNA repair protein Mms21, putative
5G08660	2.997	0.002	nuclear protein Qri2/Nse4, putative
5G11430	2.892	0.002	quinone oxidoreductase, putative
6G14140	2.612	0.003	RTA1 domain protein, putative
3G04070	2.579	0.001	bZIP transcription factor HacA
3G14230	2.572	0.009	BAR adaptor protein RVS167, putative
2G03070	2.239	0.002	conserved hypothetical protein
1G14950	2.201	0.002	sugar 1,4-lactone oxidase, putative
7G01970	2.171	0.002	RTA1 domain protein, putative
6G03760	2.169	0.003	HhH-GPD family base excision DNA repair protein
5G01800	2.137	0.001	DNA repair protein Rad14, putative
6G02070	2.130	0.009	phospholipid-translocating P-type ATPase domain-containing protein
2G09560	2.089	0.002	mitochondrial genome maintenance protein Mgm101, putative
5G08950	0.477	0.002	RAS small monomeric GTPase (Rsr1), putative
8G07130	0.475	0.002	AhpC/TSA family thioredoxin peroxidase, putative
2G11180	0.452	0.001	developmental regulator FlbA
6G11590	0.451	0.007	4-carboxymuconolactone decarboxylase family protein
3G05770	0.450	0.008	RAS small monomeric GTPase, putative
5G04333	0.442	0.004	RNA polymerase TFIIH complex subunit Ssl1, putative
1G05800	0.410	0.001	MAP kinase kinase (Mkk2), putative
5G07860	0.392	0.002	phosphatase family protein
6G09930	0.379	0.002	bZIP transcription factor AP-1/Yap1, putative
3G12530	0.377	0.005	sensor histidine kinase/response regulator, putative
1G13250	0.374	0.004	1-phosphatidylinositol-4,5-bisphosphate phosphodiesterase Plc1, putative
2G10730	0.372	0.003	DNA polymerase alpha catalytic subunit, putative

1G15270	0.367	0.004	heat shock protein Hsp98/Hsp104/ClpA, putative
1G14680	0.362	0.003	DNA-dependent RNA polymerase II largest subunit, putative
3G06780	0.357	0.010	DNA polymerase zeta catalytic subunit, putative
3G05650	0.356	0.000	alpha,alpha-trehalose-phosphate synthase subunit Tps2, putative
6G07500	0.348	0.008	DNA excision repair protein Rad2
1G05040	0.343	0.001	protein mitochondrial targeting protein (Mas1), putative
4G08300	0.337	0.000	DNA mismatch repair protein Msh6, putative
5G13220	0.333	0.004	conserved hypothetical protein
6G12570	0.328	0.000	EH domain binding protein epsin 2
5G07880	0.309	0.003	a-pheromone receptor PreA
1G05240	0.283	0.007	PHD finger and BAH domain protein (Snt2), putative
1G16970	0.281	0.003	ADP-ribosylation factor family protein
4G10860	0.277	0.003	conserved hypothetical protein
4G06970	0.277	0.009	RAD52 DNA repair protein RADC
4G08900	0.266	0.006	CDK-activating kinase assembly factor MAT1
4G09150	0.264	0.003	ABC multidrug transporter, putative
1G10940	0.248	0.000	MAP kinase kinase kinase SskB, putative
6G12290	0.246	0.006	PH domain protein
2G04960	0.238	0.002	RecQ family helicase MusN
2G17170	0.230	0.004	Na+/H+ antiporter Nha1
2G11840	0.211	0.000	transcriptional corepressor Cyc8, putative
4G00660	0.201	0.001	sensor histidine kinase/response regulator, putative
4G06570	0.188	0.004	Ras guanine-nucleotide exchange protein, putative
5G11280	0.183	0.003	conserved hypothetical protein
3G01750	0.178	0.001	G protein coupled receptor family protein
3G10830	0.177	0.000	glutathione S-transferase GstA
7G06890	0.170	0.000	Dyp-type peroxidase family protein
8G00710	0.156	0.003	secreted antimicrobial peptide, putative
2G00200	0.131	0.001	catalase, putative
5G02430	0.118	0.004	ATP dependent DNA ligase domain protein
6G12820	0.093	0.006	MAP kinase MpkB
7G02200	0.076	0.009	meiotic recombination protein (Dmc1), putative
6G10900	0.071	0.000	UV-endonuclease UVE-1
1G05850	0.059	0.000	erythromycin esterase, putative
1G01550	0.033	0.006	high affinity zinc ion transporter, putative
4G00180	0.009	0.000	fatty acid oxygenase, putative
